# Evaluation of the antimicrobial and antibiofilm activity of *Quercus coccifera* plant leaf extract against Gram-positive and Gram-negative bacteria

**DOI:** 10.14202/vetworld.2025.1253-1261

**Published:** 2025-05-21

**Authors:** Saif Aldeen Jaber

**Affiliations:** Department of Pharmacy, Middle East University, Amman, Jordan

**Keywords:** antibiofilm, antimicrobial activity, antioxidant activity, drug discovery, phytochemical screening, *Quercus coccifera*

## Abstract

**Background and Aim:**

The escalating global threat posed by antimicrobial resistance has intensified the search for novel antimicrobial agents. Plant-derived bioactive compounds represent a promising reservoir due to their chemical diversity and efficacy against resistant pathogens. *Quercus* species, traditionally utilized in herbal medicine, have shown significant bioactive potential. However, research specifically evaluating the antimicrobial and antibiofilm properties of *Quercus coccifera* remains limited. This study aimed to investigate the phytochemical composition, antimicrobial, antibiofilm, and antioxidant activities of *Q. coccifera* leaf extracts using various extraction methods and solvents with differing polarities.

**Materials and Methods:**

*Q. coccifera* leaves were harvested, dried, and extracted using solvents of varying polarity (n-hexane, chloroform, methanol, boiled water, and microwaved water). Phytochemical profiling included tests for alkaloids, tannins, glycosides, and terpenoids. Antioxidant activity was assessed using the DPPH assay. Antimicrobial activities against Gram-positive (*Staphylococcus aureus* and *Streptococcus pneumoniae*) and Gram-negative (*Pseudomonas aeruginosa*, *Escherichia coli*, and *Brucella melitensis*) bacteria were evaluated using AlamarBlue® (Invitrogen, Glasgow, UK) assay and Minimum Inhibitory Concentration (MIC) determination. Antibiofilm activity was assessed by biofilm viability tests and Minimum Biofilm Eradication Concentration (MBEC) assays.

**Results:**

Methanolic and boiled water extracts demonstrated robust phytochemical profiles (alkaloids, tannins, glycosides, and terpenoids) and significant antioxidant activity (>90% inhibition). Antimicrobial pre-evaluation indicated superior antibacterial efficacy (>90% inhibition) of these extracts, while microwaved water extracts showed moderate activity (~75% inhibition). The methanolic and boiled water extracts exhibited potent antimicrobial effects with MIC values <30 μg/mL against all tested pathogens except *S. pneumoniae*. Similarly, these extracts effectively disrupted biofilms formed by *S. aureus* and *P. aeruginosa*, with MBEC values approximately 25 μg/mL.

**Conclusion:**

Polar solvent extracts of *Q. coccifera* leaves exhibit significant antimicrobial and antibiofilm activities, underlining their potential as novel antimicrobial agents or adjuncts to existing therapies. Future studies involving cytotoxicity evaluation and *in vivo* efficacy are essential to translate these findings into clinical applications.

## INTRODUCTION

Microbial infections caused by various resistant microorganisms have raised global concern due to the significant number of annual deaths, impacts on human quality of life, and economic burden imposed on countries [1, 2]. Both pathogenic and nonpathogenic bacteria can resist the effects of various antibiotics through mechanisms such as efflux pump formation, mutations in antibiotic target sites, or biofilm development, which restrict antibiotic penetration to bacterial colonies [3]. Consequently, pharmaceutical companies and research institutions have intensified efforts to identify new antimicrobial agents through diverse drug discovery approaches, including natural sources, combinatorial techniques, and computational methodologies [4]. Natural sources have consistently demonstrated potential as reservoirs of active compounds useful for treating emerging diseases or improving therapies for existing conditions [5]. In addition, numerous active compounds approved by the Food and Drug Administration originate directly from natural sources or are derivatives thereof [5].

Extracts derived from various plant species, particularly those belonging to the genus *Quercus*, have been traditionally employed for the treatment of multiple diseases, due to their abundant bioactive constituents such as glycosides, fatty acids, tannins, alkaloids, proteins, and terpenoids [6–10]. Prior studies have highlighted the antimicrobial activities of different *Quercus* species, including *Quercus* *infectoria*, *Quercus* *petraea*, and *Quercus* *suber*, which are largely attributed to the bioactivity of their diverse secondary metabolites. Nevertheless, research on *Quercus coccifera* remains sparse, indicating the necessity for additional studies by Munir *et al*. [11] and Sánchez-Hernández *et al*. [12] to elucidate its distinctive phytochemical composition and antimicrobial potential.

Although studies by Munir *et al*. [11] and Sánchez-Hernández *et al*. [12] have highlighted the antimicrobial efficacy of various species within the genus *Quercus*, including *Q. infectoria*, *Q. petraea*, and *Q. suber*, limited research has specifically investigated the antimicrobial properties and phytochemical composition of *Q. coccifera*. Given the increasing global health threat posed by antimicrobial-resistant microorganisms [1–3], identifying novel bioactive compounds from understudied plant species such as *Q. coccifera* could offer promising solutions. To date, detailed analyses of the secondary metabolite profile, antimicrobial effectiveness, and mechanism of action of *Q. coccifera* extracts remain inadequately explored, representing a significant gap in current knowledge.

Therefore, the aim of the present study was to investigate the antimicrobial properties and phytochemical constituents of extracts obtained from *Q. coccifera*. Specifically, this research sought to identify and characterize key bioactive secondary metabolites and to evaluate their potential efficacy against a range of resistant bacterial strains. By addressing this research gap, the study aims to contribute valuable insights that may aid the development of novel antimicrobial agents derived from natural sources.

## MATERIALS AND METHODS

### Ethical approval

The study was conducted *in vitro* on plant materials and no animal or human subjects were involved in this study so ethical approval was not necessary.

### Study period and location

This study was conducted between July 7 and September 13, 2024, at the Faculty of Pharmacy at the University of Jordan in Amman

### Materials

Plant leaves were collected on the 10^th^ of March 2024 from Amman, Jordan. Canvas bags for drying the collected leaves were purchased from a local supplier. Solvents used for extraction, including n-hexane, chloroform, methanol, and biological-grade water, were obtained from Sigma-Aldrich (Montana, US). The 96-well plates for antimicrobial assays were sourced from Thermo Fisher (Birmingham, UK). Lysogeny broth (LB), utilized as bacterial growth media, and gentamicin, used as a positive control, were obtained from Sigma-Aldrich (Montana, US). AlamarBlue®, employed for microbial cell staining, was purchased from Invitrogen (Glasgow, UK). Water extracts were dried using a SuperModulyo 20L freeze dryer from Thermo Fisher Scientific (USA). Absorbance measurements for biological and DPPH assays were carried out using a Hidex Sense plate reader.

### Plant material identification

Leaves of *Q. coccifera* were taxonomically identified before collection by Professor Jameel Allaham from the Faculty of Science at Yarmouk University, Irbid, Jordan.

### Preparation of plant extracts

Solvents of varying polarity – n-hexane, chloroform, methanol, boiled water, and microwaved water – were employed to maximize extraction of bioactive phytochemicals. Microwaved water extraction, an unconventional method, was specifically explored to assess its effectiveness in preserving heat-sensitive compounds. Organic solvent extracts (n-hexane, chloroform, and methanol) were dried under nitrogen flow, whereas water extracts were freeze-dried. The dried extracts were stored at −20°C to prevent chemical degradation.

### Phytochemical screening

Extracts were screened for the presence of alkaloids using Mayer’s and Dragendorff’s tests, as well as tannins, glycosides, and terpenoids. Furthermore, phenolic content in the extracts was evaluated through the 1,1-diphenyl-2-picrylhydrazyl (DPPH) assay as described by Jaber, 2024 [8].

### Antimicrobial activity evaluation

#### Preliminary antimicrobial activity assessment

In a 96-well plate, 90 μL aliquots containing bacterial suspensions (1 × 10^8^ CFU/mL) in LB media were treated with 10 μL of each extract (1 mg/mL) and incubated in a shaker incubator at 37°C for 16 h. Gentamicin at concentrations ranging from 0.1 to 0.01 mg/mL was used as a positive control, while 90 μL aliquots of bacterial suspensions without extracts served as negative controls. Subsequently, 10 μL AlamarBlue® (Invitrogen, Glasgow, UK) was added to each well, incubated for an additional 2 h, and absorbance values were measured using a plate reader at 560 nm excitation and 590 nm emission wavelengths. Samples were prepared in triplicate to ensure robustness of the assay [9].

#### Determination of minimum inhibitory concentration (MIC)

Based on initial antimicrobial activity results, three extracts (methanol, boiled water, and microwaved water) were selected for MIC evaluation. Serial dilutions of these extracts, ranging from 1000 to 30 μg/mL, were prepared. Then, 10 μL of each dilution was mixed with 90 μL bacterial suspensions (1 × 10^8^ CFU/mL in LB media) and incubated for 16 h at 37°C. Gentamicin, at concentrations ranging from 100 to 10 μg/mL, was used as a positive control, while untreated bacterial sus-pensions served as negative controls. Following incub-ation, 10 μL AlamarBlue® was added to each well and incubated for another 2 h. Absorbance measurements were taken at 560 nm excitation and 590 nm emission wavelengths. MIC values were calculated using GraphPad Prism 5 statistical software. Each test was conducted in triplicate to confirm assay robustness [9].

### Anti-biofilm activity evaluation

#### Preliminary anti-biofilm activity assessment

Aliquots of 90 μL bacterial suspensions (1 × 10^8^ CFU/mL) of biofilm-forming strains (*Staphylococcus aureus* and *Pseudomonas aeruginosa*) were treated with 10 μL extract solutions (1 mg/mL) in a 96-well plate and incubated for 16 h at 37°C in a shaker incubator. After incubation, wells were washed twice with 100 μL sterilized phosphate-buffered saline (PBS), refilled with 90 μL sterilized PBS, and incubated for 2 h. Absorbance values at 600 nm wavelength were recorded. Gentamicin (0.1–0.01 mg/mL) served as a positive control, while untreated bacterial suspensions acted as negative controls. Tests were conducted in triplicate to verify the robustness of results [9].

#### Determination of minimum biofilm eradication concentration (MBEC)

Selected extracts (methanol, boiled water, and microwaved water) were serially diluted to concentrations ranging from 1000 to 30 μg/mL. Each dilution (10 μL) was combined with 90 μL suspensions of *S. aureus* and *P. aeruginosa* (1 × 10^8^ CFU/mL) in 96-well plates and incubated at 37°C for 16 h. Gentamicin at concentrations ranging from 100 to 10 μg/mL was included as a positive control, while untreated bacterial suspensions served as negative controls. Post-incubation, wells were washed twice with 100 μL PBS, then refilled with 90 μL PBS, and absorbance was measured at 600 nm. MBEC values were calculated using GraphPad Prism 5 software. Triplicates were prepared for all samples to confirm assay reliability [9].

### Statistical analysis

Data generated from antimicrobial and anti-biofilm assays were presented as mean values ± standard deviation. All experiments were conducted independently in triplicate to ensure data reproducibility and robustness.

Normality of data distribution was initially assessed using the Shapiro–Wilk test. Differences between treated groups and controls (negative and positive) for both antimicrobial and anti-biofilm assays were evaluated using one-way analysis of variance, followed by Tukey’s *post hoc* test for multiple comparisons. A probability (p) value of < 0.05 was considered statistically significant.

The MIC and MBEC values were determined through nonlinear regression analysis, utilizing dose-response curves (logarithmic concentration vs. response), employing the four-parameter logistic (4-PL) model provided by GraphPad Prism 5 software (GraphPad Software Inc., La Jolla, CA, USA). The regression analysis enabled precise estimation of MIC and MBEC values at which microbial growth and biofilm formation were significantly inhibited (≥90% inhibition).

GraphPad Prism 5 software was further employed to generate all graphical representations, ensuring clear visualization of the dose-response relationships, comparative antimicrobial efficacy, and biofilm eradication activities of the tested extracts.

The statistical methods selected allowed rigorous validation of the antimicrobial and anti-biofilm effects of the tested plant extracts, thereby ensuring reliability and reproducibility of the results reported in this study.

## RESULTS

### Plant extract yield

All solvents provided sufficient yield for subsequent biological and phytochemical analyses. Among the tested solvents, n-hexane produced the lowest amount and percentage yield after soaking the powdered plant leaves. Conversely, methanol yielded the highest extract quantity, as presented in Table 1.

**Table 1:** Plant extract yields after 100-g soaking in each used solvent.

Plant extract	Yield (g) (% yield)	Plant extract	Yield (g) (% yield)
n-hexane	1.1 (1.1)	Chloroform	1.6 (1.6)
Methanol	25.1 (25.1)	Boiled water	7.1 (7.1)
Microwaved water	3.7 (3.7)		

### Phytochemical composition and antioxidant activity

Phytochemical screening results of the plant extracts, summarized in Table 2, showed distinct compound profiles. The n-hexane extract tested negative for alkaloids, tannins, glycosides, and terpenoids after 1 h of appropriate tests. Alkaloids were detected exclusively in the methanol and boiled water extracts within 5–10 min. Tannins were identified in all extracts except n-hexane and appeared between 5- and 15-min. Glycosides were present in methanol and both water extracts, appearing within 5–15 min. Terpenoids appeared rapidly (within 5 min) in the methanol extract and later (10–15 min) in the chloroform extract.

**Table 2:** Chemical compositions of selected plant leaves.

Extract	Chemical class			

	Alkaloid	Tannins	Glycosides	Terpenoids
n-hexane	-	-	-	-
Chloroform	-	+	-	+
Methanol	++	++	+	+++
Boiled water	++	+	++	-
Microwaved water	-	+	+	-

+ = Indicates the presence of class after 10–15 min, ++ = Indicates the presence of chemical class after 5–10 min, +++ = Indicates the presence of chemical class before 5 min, and - = Indicates the absence of chemical class during 1 h

**Figure 1:**
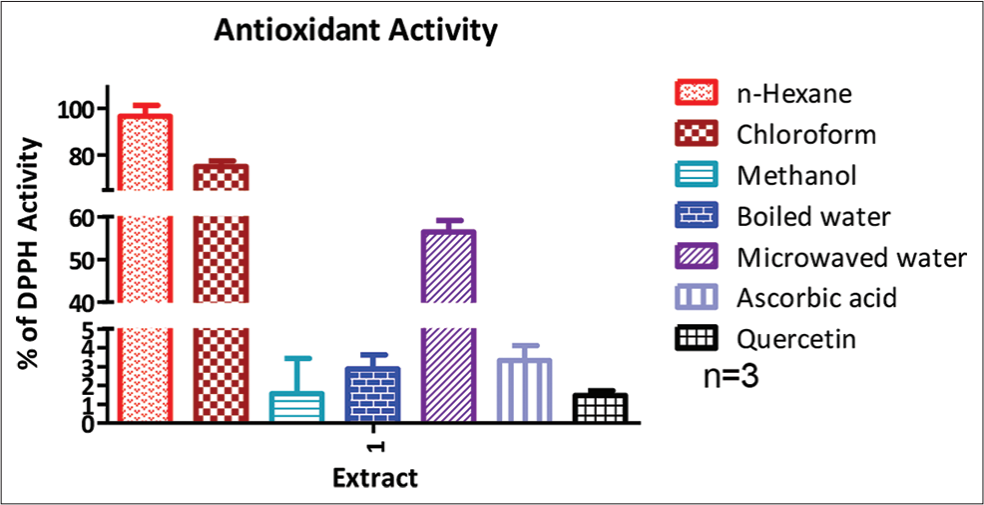
Antioxidant activity of the extracts compared with those of ascorbic acid and quercetin.

As depicted in Figure 1, the methanol and boiled water extracts exhibited strong antioxidant activity, comparable to standard antioxidants such as ascorbic acid and quercetin. This notable activity highlights the potential therapeutic role of *Q. coccifera* extracts in managing oxidative stress-related conditions and suggests the need for further biochemical and pharmacological studies. In contrast, microwaved water extract showed moderate antioxidant activity, while n-hexane and chloroform extracts demonstrated relatively weak activity.

### Antimicrobial activity evaluation

**Figure 2:**
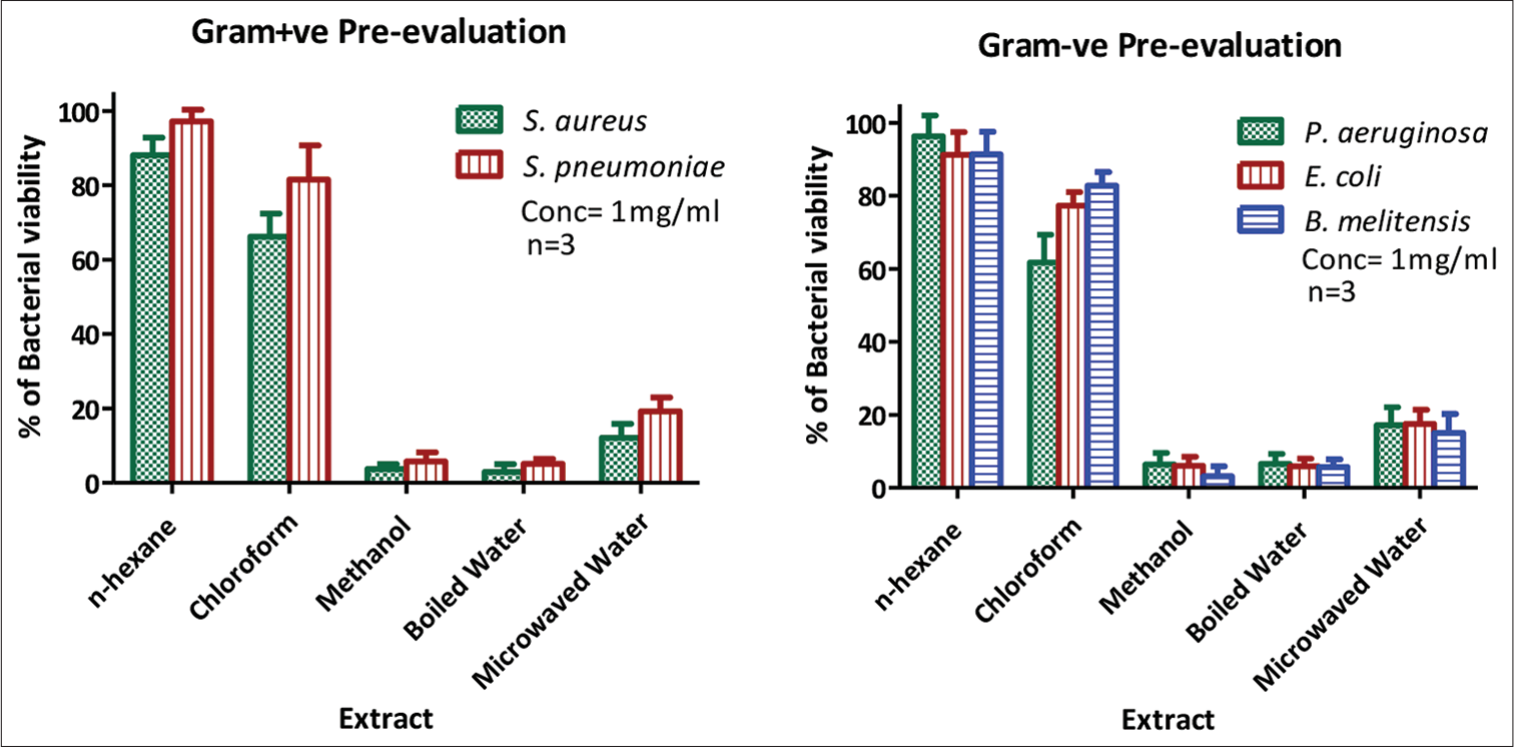
Pre-evaluation of the antimicrobial activity of 1 g/mL of *Quercus coccifera* extracts against (Gram-positive) and (Gram-negative) bacteria.

On treating selected Gram-positive and Gram-negative bacteria with each plant extract at a concentration of 1 mg/mL, extracts obtained from solvents of higher polarity (methanol, boiled water, and microwaved water) exhibited greater antimicrobial efficacy. Pre-evaluation results illustrated in Figure 2 and summarized in Table 3 showed that methanol and boiled water extracts had higher and comparable antimicrobial activities, indicated by bacterial viability rates below 5%. In comparison, the microwaved water extract demonstrated moderate effectiveness, with bacterial viability between 10% and 20%. Extracts from lower-polarity solvents (chloroform and n-hexane) resulted in relatively high bacterial viability, exceeding 60%. Based on pre-evaluation outcomes, methanol, boiled water, and microwaved water extracts were selected for subsequent IC50 assessments (Figure 2 and Table 4).

**Table 3:** Pre-evaluation results of bacteria tested using 1 mg/mL of each extract.

Gram-positive bacteria					

Bacteria	% of bacterial viability ± SD				

	n-hexane	Chloroform	Methanol	Boiled water	Microwaved water
*Staphylococcus aureus*	88.1 ± 4.7	66.2 ± 6.2	3.7 ± 1.3	2.9 ± 2.1	12.1 ± 3.8
*Staphylococcus pneumonia*	97.2 ± 3.1	81.5 ± 9.2	5.7 ± 2.5	5.1 ± 1.3	19.2 ± 3.8

**Gram-negative bacteria**					

**Bacteria**	**% of bacterial viability ± SD**				

	**n-hexane**	**Chloroform**	**Methanol**	**Boiled water**	**Microwaved water**

*Pseudomonas aeruginosa*	96.3 ± 5.7	61.7 ± 7.6	6.4 ± 3.2	6.6 ± 2.7	17.2 ± 4.9
*Escherichia coli*	91.2 ± 6.2	77.3 ± 3.7	6.1 ± 2.4	5.9 ± 2.1	17.5 ± 3.9
*Brucella melitensis*	91.3 ± 6.2	82.7 ± 3.8	3.2 ± 2.7	5.7 ± 2.1	15.1 ± 5.2

SD=Standard deviation

**Figure 3:**
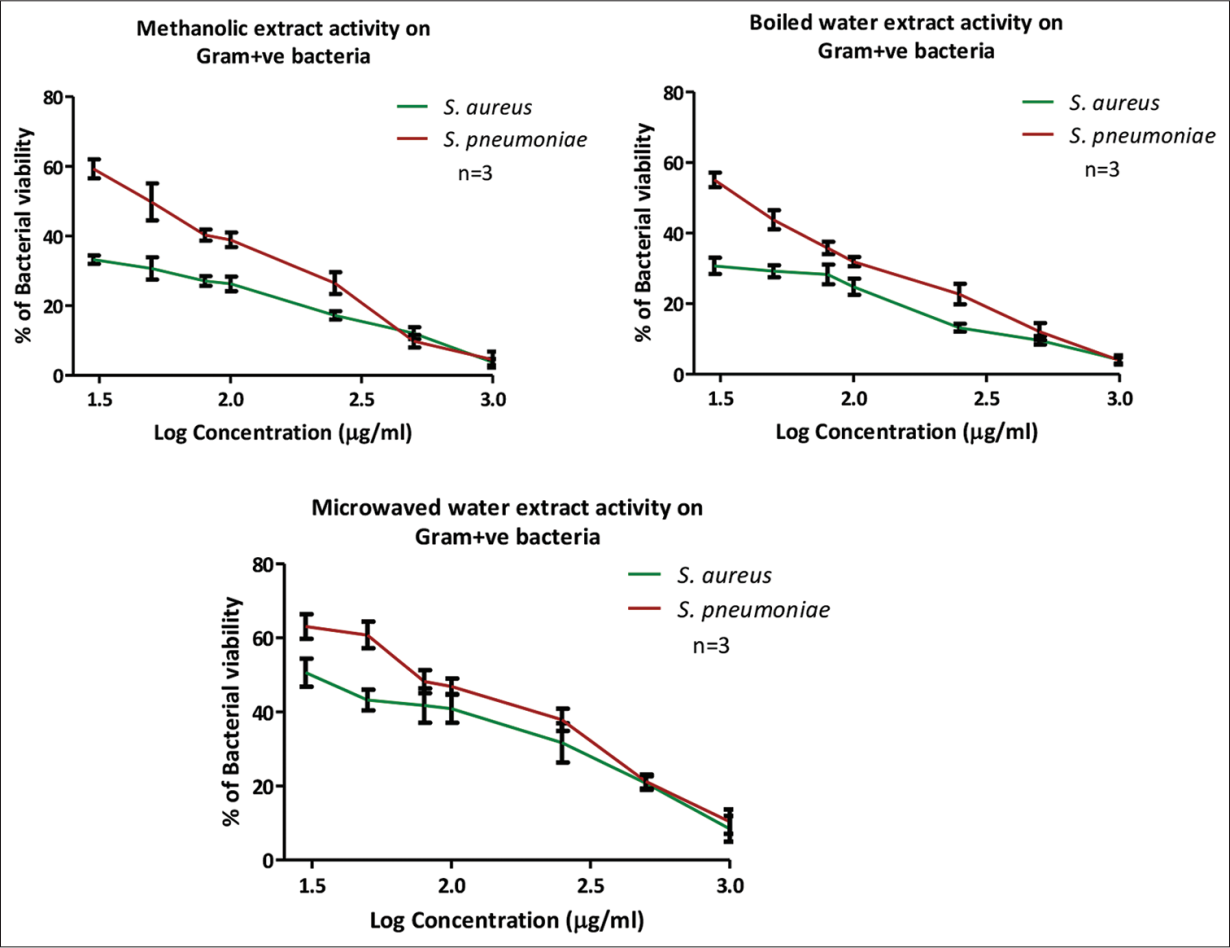
Antimicrobial activity of the active extracts (methanolic, boiled water, and microwaved water) against Gram-positive bacteria.

Detailed antimicrobial evaluation presented in Figure 3 and Table 4 indicated substantial efficacy of methanol and boiled water extracts against *S. aureus*, with MIC values below 30 μg/mL (23.51 μg/mL and 21.49 μg/mL, respectively). However, these extracts showed reduced effectiveness against *Streptococcus pneumoniae*, with MIC values ranging between 40–55 μg/mL. In addition, the microwaved water extract exhibited limited activity against these Gram-positive bacteria, yielding MIC values of 52 μg/mL (*S. aureus*) and 85 μg/mL (*S. pneumoniae*).

**Table 4:** MIC measures range for active extracts against Gram-positive bacteria.

Methanolic extract		

Results	*S. aureus*	*S. pneumonia*
MIC (μg/mL)	23.51	55.01

**Boiled water extract**		

**Results**	***S. aureus***	***S. pneumonia***

MIC (μg/mL)	21.49	43.67

**Microwaved water extract**		

**Results**	***S. aureus***	***S. pneumonia***

MIC (μg/mL)	52.73	85.73

*S. aureus*=*Staphylococcus aureus, S. pneumonia*=*Streptococcus pneumonia*, MIC=Minimum inhibitory concentration

**Figure 4:**
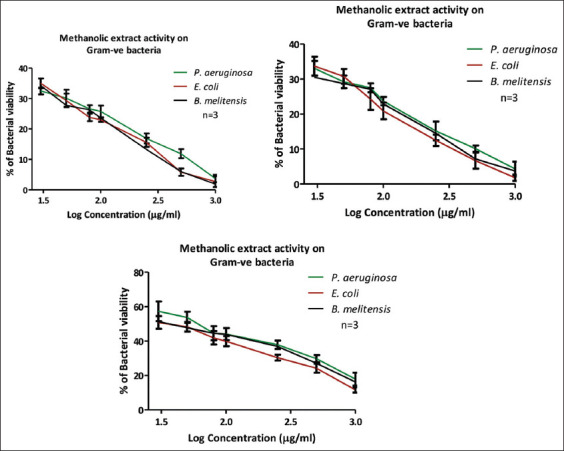
Antimicrobial activity of active extracts (methanolic, boiled water, and microwaved water) against Gram-vegetated bacteria.

Overall, antimicrobial activity was stronger against Gram-negative bacteria than Gram-positive strains. According to Figure 4 and Table 5, methanol and boiled water extracts demonstrated similar antimicrobial activities with MIC values consistently below 23 μg/mL against all three tested Gram-negative bacteria, with particularly notable efficacy against Brucella melitensis. Conversely, the microwaved water extract displayed MIC values between 56 and 76 μg/mL, showing higher efficacy specifically against *Escherichia coli*.

**Table 5:** IC_50_ range of active extracts against Gram-vegetated bacteria.

Methanolic extract			

Results	*P. aeruginosa*	*E. coli*	*B. melitensis*
MIC (μg/mL)	22.76	21.60	21.34

**Boiled water extract**			

**Results**	***P. aeruginosa***	***E. coli***	***B. melitensis***

MIC (μg/mL)	22.17	20.95	20.57

**Microwaved water extract**			

**Results**	***P. aeruginosa***	***E. coli***	***B. melitensis***

MIC (μg/mL)	75.78	56.31	65.02

MIC=Minimum inhibitory concentration, *P. aeruginosa=Pseudomonas aeruginosa, E. coli=Escherichia coli, B. melitensis=Brucella melitensis*

### Anti-biofilm activity evaluation

**Figure 5:**
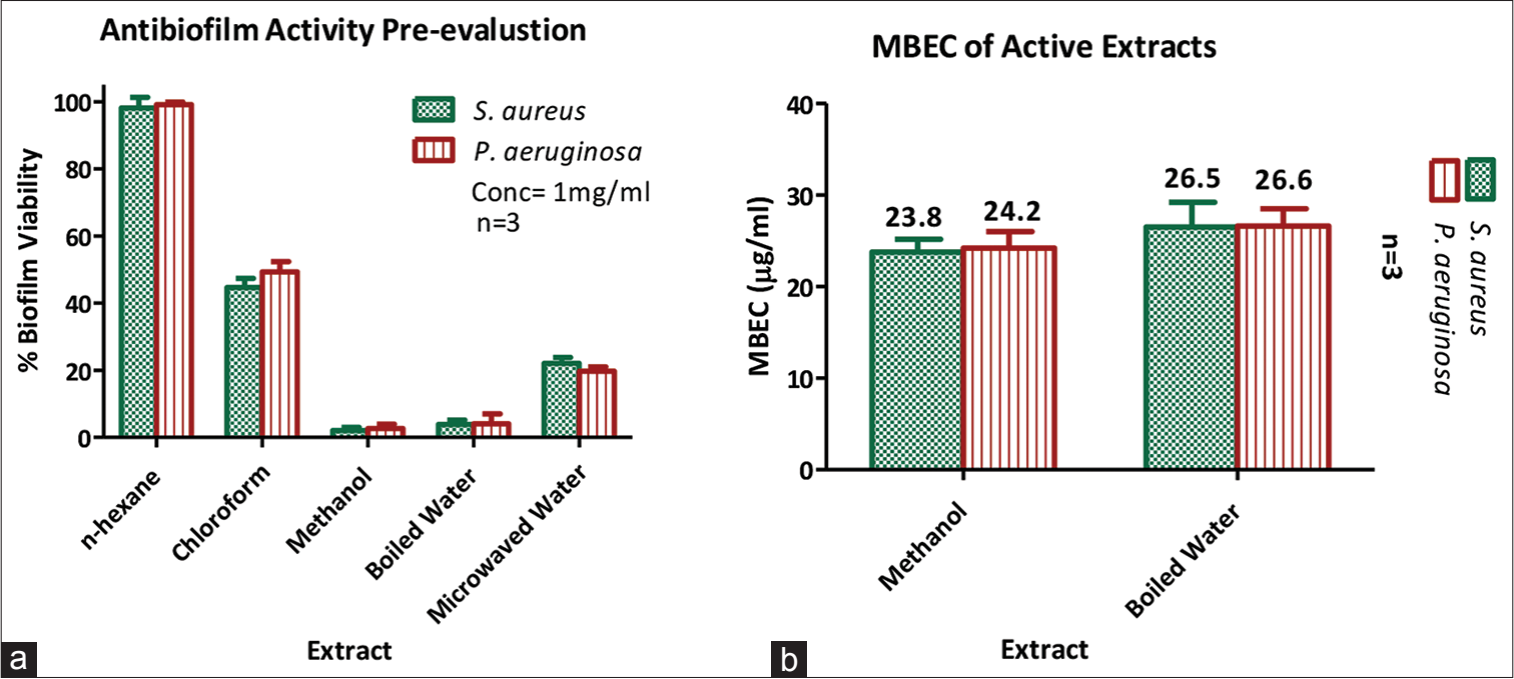
Antibiofilm activity of plant extracts. (a) Antibiofilm pre-evaluation, and (b) MBEC measuring of the active extracts.

Evaluation of anti-biofilm activity indicated that the n-hexane extract was ineffective at inhibiting or eradicating biofilms formed by *S. aureus* and *P. aeruginosa*. The chloroform extract exhibited moderate anti-biofilm activity, resulting in biofilm viability between 40% and 50%. Extracts from higher-polarity solvents, particularly the methanolic extract, showed substantial anti-biofilm properties with biofilm viability below 20%, as shown in Figure 5a and b and Table 6.

**Table 6:** Pre-evaluation of antibiofilm activity.

Bacteria	% biofilm viability of crude extracts ± SD				

	n-hexane	Chloroform	Methanol	Boiled water	Microwaved water
*Staphylococcus aureus*	98.1 ± 3.2	44.7 ± 2.7	2.1 ± 0.9	3.9 ± 1.3	22.1 ± 1.7
*Pseudomonas aeruginosa*	99.1 ± 0.8	49.3 ± 3.1	2.7 ± 1.3	4.1 ± 2.9	19.7 ± 1.3

SD=Standard deviation

Furthermore, serial dilutions of methanol and boiled water extracts effectively disrupted biofilm formation by these bacteria. MBEC values for these extracts ranged between 23.8–24.2 μg/mL (methanol) and 26.5–26.6 μg/mL (boiled water), further validating their potential for anti-biofilm applications.

## DISCUSSION

Naturally derived chemical compounds have garnered substantial interest from research institutions and pharmaceutical industries due to their demonstrated efficacy against various bacterial infections [13, 14]. This growing attention is largely attributed to the increasing resistance of pathogenic bacteria to conventional antimicrobials, primarily driven by the widespread misuse and overuse of antibiotics [15].

Several *Quercus* species have been reported to possess diverse classes of bioactive compounds with notable antimicrobial activity, including alkaloids, glycosides, tannins, and terpenoids [8, 15, 16]. These phytochemical-rich extracts represent promising reservoirs for isolating potent antimicrobial agents capable of overcoming microbial resistance, which has far-reaching implications for both public health and economic sustainability [15, 17].

Phytochemical screening of *Q. coccifera* leaves collected in March revealed that methanol and boiled water extracts contained high concentrations of key antimicrobial chemical classes, with the exception of terpenoids in the boiled water extract. Accordingly, these extracts were anticipated to exhibit strong antimicrobial activity. In contrast, extracts derived from microwaved water and chloroform were expected to display moderate activity, while the n-hexane extract was predicted to show minimal or negligible antimicrobial properties due to the absence or low abundance of essential phytochemicals. Furthermore, the seasonal timing of plant material collection significantly influenced compound concentration, as the phytochemical profile differed from that of samples collected in May 2023 [7, 8].

Antioxidant evaluations using the DPPH assay further supported the potential of methanol and boiled water extracts, with both showing inhibition rates exceeding 95%. Although both extracts demonstrated comparable antioxidant activities, the boiled water extract exhibited marginally superior performance [8]. Compounds with antioxidant properties have frequently been reported to exert broad-spectrum antimicrobial effects against both Gram-positive and Gram-negative bacteria [18].

Preliminary antimicrobial assays at 1 mg/mL revealed that polar extracts – specifically methanol and boiled water – achieved over 90% inhibition of bacterial growth. Microwaved water extract demonstrated moderate efficacy, inhibiting approximately 80% of both bacterial groups. These findings align with prior chemical and biological characterizations of various *Quercus* species, where polar extracts from different plant parts were shown to possess heightened antimicrobial potential due to higher concentrations of active constituents [19].

MIC assays of extracts exhibiting strong initial antimicrobial activity revealed that methanol and boiled water extracts had lower MIC values against *S. aureus* (<25 μg/mL) compared to the microwaved water extract. However, antimicrobial activity was somewhat reduced against *S. pneumoniae*, with MIC values approximating 50 μg/mL. These MIC values for *Q. coccifera* extracts were substantially lower than those reported for other *Quercus* species such as *Q. rubra* and *Q. infectoria*, which exhibited MICs of 300 μg/mL and 70 μg/mL, respectively [20].

Similarly, methanol and boiled water extracts displayed superior activity against *S. pneumoniae* when compared to other *Quercus* species, such as *Q. rubra*, which presented MICs as high as 600 μg/mL [20, 21]. Against Gram-negative bacteria, methanol and boiled water extracts again demonstrated strong activity, with MICs below 25 μg/mL. In comparison, the microwaved water extract yielded MIC values between 56 and 76 μg/mL. These results were again more favorable than those recorded for *Q. rubra* extracts, which exhibited minimum MIC values of 600 μg/mL against Gram-negative strains [21].

All extracts were initially screened against biofilm-forming *S. aureus* and *P. aeruginosa* to assess their potential anti-biofilm properties in combating biofilm-associated antimicrobial resistance. Consistent with their antimicrobial profiles, methanol and boiled water extracts demons-trated the highest biofilm inhibition rates, exceeding 95%. These were followed in descending order by microwaved water, chloroform, and n-hexane extracts. The pronounced anti-biofilm activity of polar extracts is likely attributed to their ability to disrupt intercellular matrix components or interfere with quorum sensing and intracellular signaling within bacterial communities [22, 23].

Given their superior performance, methanol and boiled water extracts were selected for MBEC determination. Both extracts exhibited low MBEC values, averaging around 25 μg/mL. Notably, these values were significantly lower than those reported for other *Quercus* species such as *Q. rubra* and *Q. infectoria*, which recorded MBECs exceeding 1000 μg/mL [20, 24]. The ability to inhibit biofilm formation presents a strategic advantage in reducing bacterial resistance and improving therapeutic outcomes for antimicrobial treatments [25].

## CONCLUSION

This study highlights the promising antimicrobial and antioxidant potential of *Q. coccifera* leaf extracts, particularly those obtained using polar solvents such as methanol and boiled water. Among the various extracts tested, methanol and boiled water extracts consistently exhibited superior antimicrobial activity, with MIC values below 25 μg/mL against *S. aureus*, *B. melitensis*, and *E. coli*, and slightly higher values against *S. pneumoniae*. Furthermore, both extracts demonstrated strong anti-biofilm properties, achieving MBEC values in the range of 23.8–26.6 μg/mL, significantly outperforming extracts from other *Quercus* species such as *Q. rubra* and *Q. infectoria*.

Phytochemical analysis revealed that the antimicrobial efficacy of the methanolic and boiled water extracts could be attributed to the presence of key bioactive compounds, including alkaloids, tannins, glycosides, and terpenoids. The strong antioxidant activity, with DPPH inhibition exceeding 95%, further supports the therapeutic potential of *Q. coccifera* in managing oxidative stress-related infections.

These findings underscore the potential of *Q. coccifera* as a valuable source of plant-based antimicrobial agents. The low MIC and MBEC values suggest that these extracts could be further explored as candidates for pharmaceutical development, particularly for treating drug-resistant bacterial infections and biofilm-associated chronic conditions.

The study employed a comprehensive approach combining phytochemical screening, antioxidant assessment, and a two-tier antimicrobial evaluation (planktonic and biofilm forms), thereby ensuring a robust understanding of the biological efficacy of *Q. coccifera* extracts. In addition, direct comparison with previously published data on other *Quercus* spec-ies enhanced the relevance and contextual value of the results.

Despite its strengths, the study is limited by its *in vitro* design, which may not fully reflect *in vivo* efficacy, pharmacokinetics, or toxicity profiles. The precise mechanisms of antimicrobial and anti-biofilm action were not elucidated, and the study did not isolate or characterize individual active compounds from the crude extracts.

Further investigations should focus on the isola-tion, structural elucidation, and mechanistic studies of the active phytoconstituents responsible for the obser-ved bioactivities. *In vivo* studies and clinical evalua- tions are necessary to validate the therapeutic potential of these extracts. In addition, exploring synergistic effects with existing antibiotics could offer novel combinatory therapies to combat antimicrobial resistance.

In conclusion, *Q. coccifera* leaf extracts, particularly those derived from methanol and boiled water, represent a promising natural source of antimicrobial and anti-biofilm agents, meriting continued research toward pharmaceutical development.

## AUTHOR’S CONTRIBUTIONS

SAJ: Designed and conducted the study, interpreted the results, and drafted the manuscript. The author has read, reviewed, and approved the final manuscript.
